# Make the Foundation Firm

**Published:** 1866-09

**Authors:** G. W. Decamp


### MAKE THE FOUNDATION FIRM!

BY G. W. DECAMP, D. D. S.

DENTAL COSMOS, vol. 7, page 652: "*Resolved*, That we individually recognize it to be our duty to earnestly advise all who are seeking to enter our profession to pursue a regular course of study in some dental college, in connection with private instruction."

This is good; but is it sufficient? Should there not first be a thorough education, so that this cap stone may rest on a fit foundation? Let us examine the walls, and see if they are strong enough to bear this weight, that we may build a durable temple, its halls and arches and towers remaining in beauty.

In rearing the temple of mental excellence, as in the material world, there must first be a preparation. The heart is the head of the corner. Its preparation is of God. Without it our structure will be hurled to the ground by every storm. If the heart be pure, the man will be full of light.

Each piece must be shaped for its place, or the whole will be weak. This is done alone by the Genius, Education. Knowledge does not comprise all that is contained in the term "Education." True and worthy motives are to be inspired, the feelings to be restrained, the passions to be conquered, morality to be inculcated under all circumstances, and the fear of God always abiding in the heart. "The fear of the Lord is the beginning of wisdom." All these are included in "Education."

That these may be, the first impressions made must be pure. If these be good the mind will advance. Yawning chasms, or burning mountains, or boiling seas, or heaving valleys, can not stop it. Onward and upward it goes to the goal of its ambition. It looks with longing on the distant hights; with solicitude and perseverance it struggles up the steps. Solici tude is characteristic of a strong mind. Man is conceived, nourished, born, acts his part, and dies, in solicitude.

If a young man persevere, I ask not the measure of his brain. He has a sure passport to success. If the heavens should fall, they would find him on the way to mental excellence. Throw away the false idea that "circumstances make the man." Man is the builder of his own fortune. There are no beds of ease on which to carry drones to victory. Difficulties must come, as smoke flies upward; that is by a law of nature. They are blessings. They stimulate to greater action, and teach the mind to conquer. By them its powers are developed.

What makes the Indian swift on foot, but the fleetness of the deer? The eye of the hunter is keen, because of the shrewdness of the game. The weight of the hammer gives strength to the blacksmith's arm. Who are the deepest reasoners, but those who have dared wrestle with great difficulties. What would the foal of the racer know of its power, if kept with the common herd, destined for the yoke? Difficulties arouse to action. Actions make the man.

That the mind may grow, it must be nourished. Man would not thrive if fed on apples. He needs a variety. The sciences are the food of the mind. Restrict it to any one, and it will become weak. The eye of the criminal confined to his cell is not as strong as that of the sailor, who looks from the mast-head.

It is not only necessary for all who intend to practice the science of Dentistry to be learned in what directly pertains to it, but they must be *educated.*

Some may object, by saying that it is not necessary. They will tell me that our fathers were not learned; that some of the brightest stars of the present entered the profession illiterate; and, with a great degree of satisfaction, fold their hands for a *little more* sleep. Thus would they block the wheels of progress, and banish us to everlasting ignorance.

Should we live in log huts because our fathers did? Should we refuse to improve our advantages because our fathers had them not? No! No! In the name of our God we cry, No! Deny me any pleasure, but deny me not the use of what I have. Humanity shudders at the thought. Talent is adverse to ignorance. Our advantages are greater than our fathers'; and although some of them, by herculean efforts, stand among the clouds, many worship at the shrine of ignorance. Our fathers improved what they had; we should do likewise. If we do, we shall be *better* than *they*. We must be *wiser* or *more ignorant*.

The position and respect which the title of D. D. S. is intended to command should urge a thorough education. If we respect ourselves we must aim at mental and moral excellence. Our land is covered with colleges, where the young may learn to think. There is no excuse for ignorance, when good books, the products of the richest minds and the labor of many years, are within the reach of all. If a thorough preparation is made, those who enter our profession may bless it, bless humanity, and be blessed of God.

That we may be, *Resolved*, That we individually recognize it to be our duty to earnestly constrain all who intend to enter our profession to educate themselves, so that they may be prepared to pursue a regular course of study in some dental college, in connection with private instruction. This is my hope.

